# Creutzfeldt-Jakob Disease Presenting with Abducens Nerve Palsy

**DOI:** 10.7759/cureus.5564

**Published:** 2019-09-04

**Authors:** Christopher M Anthony, Gregory B Giles, Grant A Justin, Marissa L Wedel, Aaron D Grant

**Affiliations:** 1 Ophthalmology, Western University of Health Sciences, Lebanon, USA; 2 Ophthalmology, Brooke Army Medical Center, San Antonio, USA; 3 Ophthalmology, Wilford Hall Ambulatory Surgical Center, San Antonio, USA

**Keywords:** creutzfeldt-jakob disease (cjd), ophthalmology, cranial nerve six palsy, abducens nerve

## Abstract

Creutzfeldt-Jakob disease (CJD) is a rare neurodegenerative disorder with characteristic clinical and diagnostic features. We describe the unusual case of an elderly man who presented to our ophthalmology clinic with horizontal diplopia secondary to an abducens nerve (cranial nerve six) palsy and was subsequently diagnosed with CJD. Given the non-treatable nature of this disease, ophthalmologic management goals included symptomatic relief and quality of life improvement. Precautions related to the ophthalmologic management of CJD have also been addressed in this case report.

## Introduction

Creutzfeldt-Jakob disease (CJD) is a rare, rapidly progressive, transmissible, and uniformly fatal neurodegenerative disorder resulting in spongiform encephalopathy with an estimated incidence of 1 to 1.5 cases per one million population annually [1]. Although numerous types and subtypes of this disease exist, each is believed to be caused by an abnormal isoform of a cellular glycoprotein known as prion (proteinaceous infectious particles) protein. The common ophthalmologic manifestations of this disease include worsening visual acuity, blurry vision, visual distortion, changes in-depth perception, tunnel vision, visual field loss, visual hallucinations, cortical blindness, diplopia, supranuclear palsies, and complete loss of vision, but rarely abducens nerve palsy [2]. Ophthalmologists should be aware of abducens nerve palsy as a possible early manifestation of CJD and act accordingly. We herein report the clinical presentation, diagnosis, and ophthalmologic management of a patient with CJD of probable spontaneous etiology presenting with abducens nerve palsy.

## Case presentation

A 60-year-old male with a history of diabetes, hypertension, hyperlipidemia, hypothyroidism, and photorefractive keratectomy surgery (PRK) initially presented to the ophthalmology clinic complaining of acute-onset horizontal double vision. On review of systems, the patient also reported gait changes, instability, aphasia, forgetfulness, and weight loss of one month’s duration without reported episodes of metabolic derangement, toxic exposure, or infection. 

On initial physical examination, the patient was found to have visual acuity of 20/40 in the right eye (pinhole to 20/20) and 20/30 in the left eye (pinhole to 20/25), pressure in both eyes of 15 mmHg, and no afferent pupillary defect. Confrontational visual fields were full along with full-color plates. Sensorimotor measurements included 8 diopters (D) of esotropia (ET) in primary gaze, 6D of ET in left gaze, 10D of ET in right gaze, 2D of ET in upgaze, and 6D of ET in downgaze, with a restriction of 1 on a 0-4 scale noted in right gaze. On slit-lamp examination, the patient was noted to have 1+ nuclear sclerosing cataracts in both eyes with a cup-to-disc ratio of 0.3 in both eyes and no abnormalities on dilated fundus exam. 

**Figure 1:**
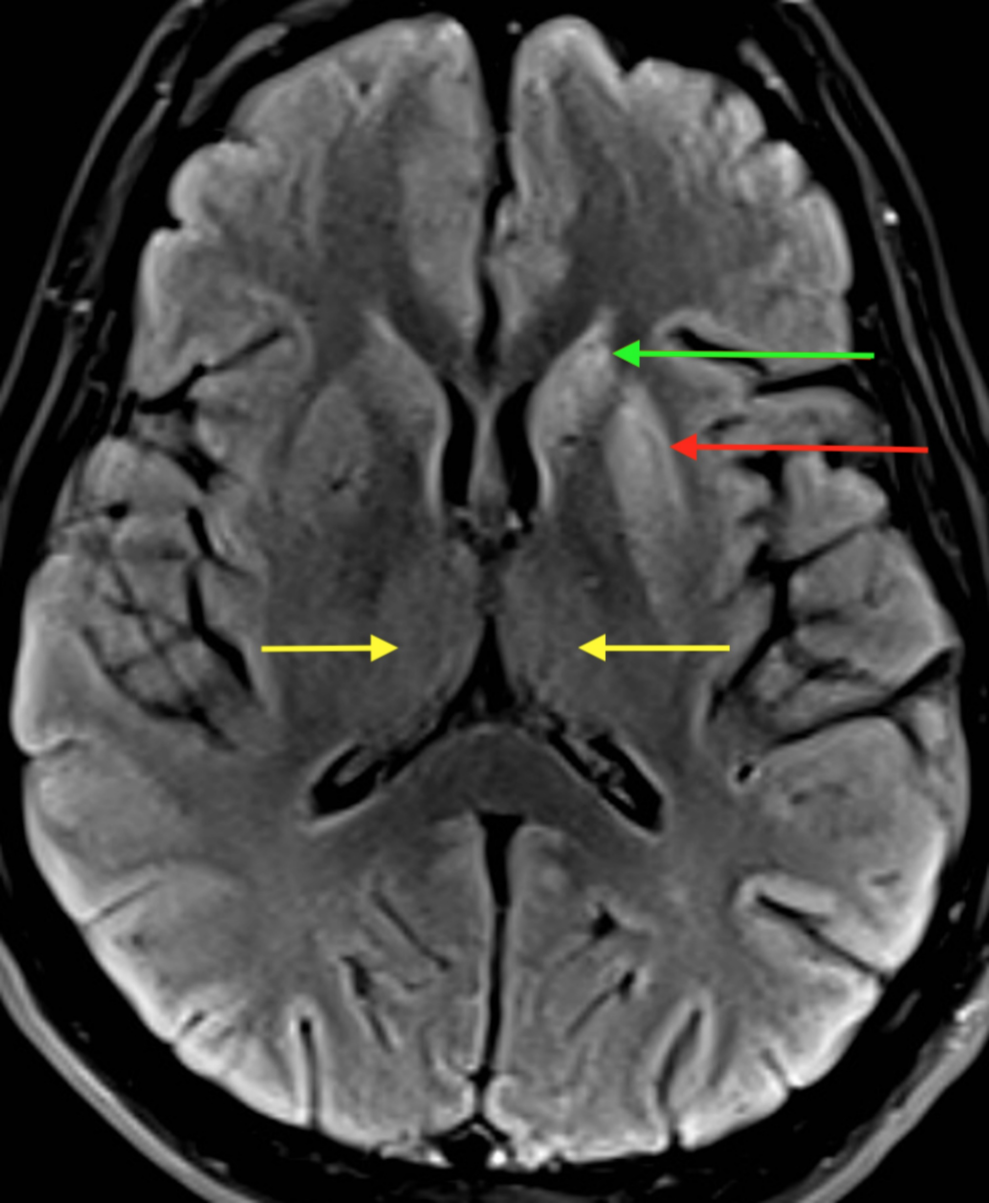
T2/FLAIR MRI axial section showing hyperintense signals in the left caudate (green arrow), putamen (red arrow), and dorsomedial thalami (yellow arrows) consistent with CJD FLAIR, fluid-attenuated inversion recovery; MRI, magnetic resonance imaging; CJD, Creutzfeldt-Jakob disease

An MRI was obtained less than one week later which showed diffusion restriction and T2/FLAIR hyperintense signal in the left greater than right caudate and putamen and dorsomedial thalami (Figure 1). A small focus of diffusion restriction was noted in the left globus pallidus as well as diffusion restriction involving the cortex of the left cingulate gyrus, posterior portions of the left superior frontal gyrus, left insular cortex, left frontal opercular cortex, and left precentral gyrus. A few punctate foci of nonspecific T2 hyperintensity were seen within the cerebral white matter. Differential diagnosis based on the MRI results included CJD, hypoxic-ischemic encephalopathy, or osmotic demyelination.

Given the MRI findings in the setting of rapidly progressive dementia and cerebellar signs, the patient was admitted for an expedited workup which included repeated advanced neuro-imaging, electroencephalogram (EEG), and CSF studies. The initial MRI findings were confirmed, EEG findings were unremarkable without any periodic sharp wave complexes, and the patient was found to have Creutzfeldt-Jakob 14-3-3 protein and RT-QuIC in his cerebral spinal fluid two weeks after initial presentation. In a recent study, Creutzfeldt-Jakob 14-3-3 protein was estimated to have a sensitive and specificity of 92% and 80%, respectively, in detecting CJD. However, false-positive results occur in numerous disorders including acute stroke, encephalitis, and other dementing disorders [3]. Based on the Centers for Disease Control and Prevention (CDC) criteria, the patient was diagnosed with CJD of probable spontaneous etiology. Although other variants of CJD, including heritable and acquired, were considered as possible etiologies, the spontaneous variant was determined to be the most likely as it accounts for approximately 85% of cases worldwide and the patient had neither a family history of similar symptoms nor had risk factors for the acquired variant. 

Ophthalmologic follow-up was completed five weeks after initial presentation with the patient complaining of worsening horizontal double vision in addition to the previously mentioned neurological symptoms. Physical exam findings, including persistent abducens nerve palsy, were similar to those found on initial presentation with the exception of a marked rapid horizontal beating pattern of both eyes which lacked a fast or slow component. Neuro-ophthalmology was consulted to evaluate for prism correction of diplopia with the goal of providing symptomatic relief and quality of life improvement, but the patient expired prior to the appointment. 

## Discussion

Based on the current literature, visual disturbance is evident in 10% to 20% of cases of sporadic CJD [2]. Armstrong et al. states “the most commonly reported visual symptoms include diplopia, supranuclear palsies, complex visual disturbances, homonymous visual field defects, hallucinations and cortical blindness [4].” In particular, oculomotor disturbances have been reported to occur at some point in 20% to 30% of patients with sporadic CJD, but according to Lueck et. al “many reports use terms which do not allow further comment, such as 'difficulty with conjugate eye movements', ‘erratic eye movements’, 'fluttering ocular movements', or 'ophthalmoplegia.” Rarely is a concrete case of abducens nerve palsy secondary to CJD reported [2]. This case is unique and of value because, to the best of our knowledge, there are exceedingly few published case reports specifically regarding abducens nerve palsy as an early manifestation of CJD. 

Although abducens nerve palsy is the single most common extraocular muscle palsy and the differential of its causes is sizeable, our case shows that it should be counted as a valuable piece of information in raising early clinical suspicion for CJD when combined with rapid onset cognitive and functional impairment. Despite the fact that our patient had classical vascular risk factors for cranial nerve palsy, including diabetes, hypertension, and hyperlipidemia, the acute onset of the patient’s neurological deficits in conjunction with neuroimaging and 14-3-3 CSF protein strongly suggests CJD as the cause of his abducens nerve palsy. Differential diagnosis also included acute stroke, encephalitis, and other dementing disorders. Maintaining awareness of ophthalmologic findings such as abducens nerve palsy can allow for expeditious diagnosis of CJD and potentially improve patients’ quality of life by allowing for planning of palliative care options and end-of-life arrangements. 

Early diagnosis of CJD is not only beneficial for the afflicted patient but also potentially life-saving for otherwise healthy patients undergoing routine ophthalmologic procedures, such as applanation, who may be exposed to ophthalmic instruments used on patients with undiagnosed CJD. The transmissible proteins responsible for CJD are known to accumulate in corneal dendritic cells which help to mediate the immune response within the anterior chamber. According to Armstrong “the presence of these cells in the cornea has raised the possibility of transmission between patients via optical devices that contact the eye,” but the risk of transmission is exceedingly low [4-5]. Ophthalmologists should be aware of this theoretical risk and act accordingly by using appropriate cleaning and disinfection protocols and identifying and quarantining instruments used on infected or potentially infected patients [5].

## Conclusions

Although abducens nerve palsy is a common extraocular muscle palsy and the differential of its causes is sizeable, it should be counted as a valuable piece of information in raising early clinical suspicion for CJD when combined with rapid onset cognitive and functional impairment. Ophthalmologists, especially those working with limited resources or in areas known to have a higher prevalence of CJD, should be aware of this risk, develop protocols for effectively identifying and quarantining instruments used on infected or potentially infected patients, and apply appropriate disinfection measures. 
